# Apobec3A Deamination Functions Are Involved in Antagonizing Efficient Human Adenovirus Replication and Gene Expression

**DOI:** 10.1128/mbio.03478-22

**Published:** 2023-05-08

**Authors:** Lilian Göttig, Christina Weiß, Miona Stubbe, Lisa Hanrieder, Samuel Hofmann, Alessandro Grodziecki, Daniela Stadler, Arnaud Carpentier, Ulrike Protzer, Sabrina Schreiner

**Affiliations:** a Institute of Virology, School of Medicine, Technical University of Munich, Munich, Germany; b Institute of Virology, Hannover Medical School, Hannover, Germany; c German Center for Infection Research (DZIF), Munich, Germany; d Cluster of Excellence RESIST (Resolving Infection Susceptibility; EXC 2155), Hannover Medical School, Hannover, Germany; e Institute of Experimental Virology, Twincore, Hannover, Germany; f Institute of Virology, Helmholtz Zentrum München, Munich, Germany; The University of Texas Health Science Center at San Antonio; Columbia University Medical Center

**Keywords:** HAdV, adenovirus, A3A, Apobec, SUMO, deamination, SUMOylation, virus host interaction

## Abstract

Apobec3A is involved in the antiviral host defense, targeting nuclear DNA, introducing point mutations, and thereby activating DNA damage response (DDR). Here, we found a significant upregulation of Apobec3A during HAdV infection, including Apobec3A protein stabilization mediated by the viral proteins E1B-55K and E4orf6, which subsequently limited HAdV replication and most likely involved a deaminase-dependent mechanism. The transient silencing of Apobec3A enhanced adenoviral replication. HAdV triggered Apobec3A dimer formation and enhanced activity to repress the virus. Apobec3A decreased E2A SUMOylation and interfered with viral replication centers. A comparative sequence analysis revealed that HAdV types A, C, and F may have evolved a strategy to escape Apobec3A-mediated deamination via reduced frequencies of TC dinucleotides within the viral genome. Although viral components induce major changes within infected cells to support lytic life cycles, our findings demonstrate that host Apobec3A-mediated restriction limits virus replication, albeit that HAdV may have evolved to escape this restriction. This allows for novel insights into the HAdV/host-cell interplay, which broaden the current view of how a host cell can limit HAdV infection.

## INTRODUCTION

Human adenoviruses (HAdV), which were first isolated from human adenoids in 1953, are small, nonenveloped icosahedral viruses that consist of a double-stranded linear genome (1–3). To date, more than 95 different HAdV have been discovered, and these can be further classified into species A to G (4–6). An adenoviral infection can cause many different diseases, including conjunctivitis, respiratory diseases, and gastroenteritis. In immunocompetent individuals, adenoviral infections lead to acute, self-limiting diseases, whereas in immunosuppressed patients, an infection can cause severe outcomes with high mortality and morbidity rates (7–9). Furthermore, new evolving strains, such as HAdV14p1, were found to be lethal, even in healthy individuals, emphasizing the need for new therapeutic strategies (10). So far, no therapy is available, and diseases are only treated symptomatically (11). To find new treatment options, it is necessary to investigate and understand early virus-host interactions.

HAdV have evolved several mechanisms by which to suppress the activation of the DNA damage response (DDR) to enable efficient replication. The most important mechanism is the E3 ubiquitin ligase complex, which is formed by the two early viral proteins E1B-55K and E4orf6. Together with several cellular factors, such as ElonginB/C, Cullin2/5, and Rbx1, the virus targets host substrates for ubiquitinylation and subsequent proteasomal degradation. This suppression of the DDR is necessary for HAdV to enable efficient replication and progeny virus production (12–17).

A cellular factor that is known to activate the DDR is the human apolipoprotein-B mRNA-editing catalytic polypetide-like 3A protein, namely, Apobec3A (18), which is a member of the family of Apobec cytidine deaminases. In general, all 11 Apobecs contain a conserved, zinc-dependent deaminase motif (19) and bind DNA and/or RNA to target nucleotide sequences. Most members of this family induce the deamination of cytosines to uracils, which is then followed by the excision of uracil residues, introducing breaks, nicks, and point mutations within the sequence (20–22). For instance, the subfamily Apobec3, and, specifically, its members Apobec3C, D, F, G, and H, mutates the viral genome of HIV-1 during infection. To counteract this antiviral mechanism, the HIV-1 accessory protein Vif binds to and recruits these proteins to an E3 ubiquitin ligase for ubiquitinylation and proteasomal degradation (23, 24).

Apobec3A is thought to be one of the most active Apobec proteins, and it was shown to inhibit HIV, parvoviruses, HTLV, and HPV (25–29). It also plays an important role in degrading the persistent HBV genome, the covalently closed circular DNA (cccDNA) (27). In most cases, Apobec3A deaminates viral genomes or foreign DNA, thereby acting in an antiviral manner. However, the parvovirus infection is inhibited by Apobec3A via a deaminase-independent and currently unknown mechanism (25, 26, 28–30). For antiviral defense, endogenous Apobec3A can translocate to the nucleus during viral infection (27). Transfected Apobec3A was shown to localize to both the nucleus and the cytoplasm, where it interferes with the establishment of AAV replication centers (30). Enforced by a symmetric swap of the N-terminal residues, the functional form of Apobec3A is comprised of a homodimer, which allows for specific substrate recognition and enhanced catalytic activity (31). Furthermore, Apobec3A has been proposed to play a crucial role in oncogenesis, as Apobec3A-induced mutations are increased in different cancer types (32, 33).

Since the expression of Apobec3A can lead to DNA breaks and thereby activate DDR (18), we hypothesized that Apobec3A is detrimental to HAdV, which needs to suppress DDR to replicate efficiently (34). To analyze this, we investigated the role of Apobec3A during HAdV replication. Our data showed that Apobec3A is not a target of the E3 ubiquitin ligase complex, since Apobec3A levels actually increased during HAdV infection. Apobec3A expression negatively regulated viral DNA replication, gene expression, and progeny production, which was linked to a deaminase-dependent mechanism. Apobec3A also interfered with the establishment of HAdV replication centers. During infection, our assays on endogenous Apobec3A and during overexpression revealed that Apobec3A can be stabilized at the translational level and can be upregulated at the transcriptional level, as was also the case for Apobec3A dimer formation, implying enhanced Apobec3A activity during infection. Intriguingly, we provide evidence that HAdV genes include a reduced number of TC dinucleotides, compared to human genomes and other human-pathogenic DNA viruses, suggesting a potential evolution-based strategy by which to circumvent massive Apobec3A deamination.

## RESULTS

### Apobec3A levels are elevated during HAdV infection.

**FIG 1:**
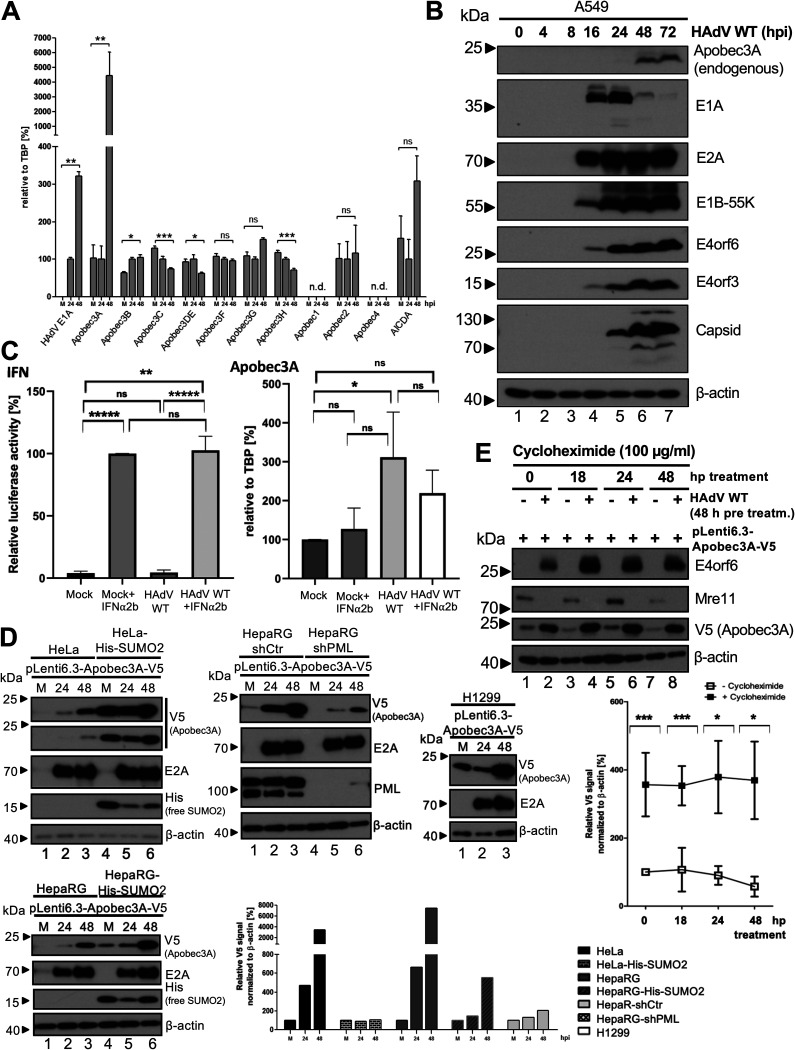
Apobec3A gene expression is stabilized during HAdV infection. (A) HepaRG cells were infected with HAdV wt (50 ffu/cell) and harvested after 0, 24, and 48 h postinfection (hpi). mRNA was isolated, and cDNA was reverse transcribed. This was followed by a qPCR analysis. The E1A transcript was used as an infection control. mRNA levels were set in relation to the housekeeping TBP mRNA levels. (B) A549 cells were infected with HAdV wt (20 ffu/cell). After harvesting the cells at the indicated time points (0 to 72 hpi), cell lysates were generated, and proteins were separated via SDS-PAGE and subjected to Western blotting. β-actin served as the loading control. The stained proteins are indicated on the right, and the molecular weights are indicated in kDa on the left. (C) A549 cells were treated with 1,000 U/mL IFN-α2b and HAdV wt (50 ffu/cell) at 24 h preinfection. The cells were harvested at 48 hpi for mRNA isolation, cDNA transcription, and qPCR analysis for Apobec3A transcription (right panel). A control set-up was treated and infected equally to harvest the IFN-secreted supernatant at 72 h post treatment. Dilution rows of the supernatant were added for 6 h to the reporter cell line HL116, which expressed a stably integrated luciferase gene under the control of the interferon-responsive promoter region 6 to 16. Dual luciferase reporter assays were performed to measure IFN secretion, and median values were calculated (left panel) (D) HeLa, HeLa-His-SUMO2, HepaRG-shCtrl, HepaRG-shPML, HepaRG, HepaRG-His-SUMO2, and H1299 cells were transfected with 5 μg pLenti6.3-Apobec3A-V5 and were infected with HAdV wt (50 ffu/cell). Cells were harvested at 24 and 48 hpi and were analyzed via Western blotting, as in panel B. Signal intensities for V5 Apobec3A were calculated in relation to the β-actin loading controls using the ImageJ program. (E) HepaRG cells were transfected with 5 μg pLenti6.3-Apobec3A-V5 and were infected with HAdV wt (50 ffu/cell). At 48 hpi, the cells were treated with 100 μg/mL cycloheximide, and this was followed by harvesting at the indicated time points, post-treatment. Whole-cell lysates were generated and analyzed via Western blotting as in panel B, additionally using an antibody against Mre11. The signal intensities for V5 Apobec3A were calculated in relation to the β-actin loading controls, using the ImageJ program. Statistical significance was assessed via an unpaired *t* test (*, *P* < 0.05; **, *P* < 0.01; ***, *P* < 0.005; ns, not significant; n.d., not detected; hpi, hours postinfection).

Since Apobec proteins possess restrictive functions against many different viruses, the effect of HAdV infection on Apobec expression was investigated by performing a qRT-PCR analysis. HepaRG cells infected with HAdV-C5 wt were harvested at 24 and 48 hours post infection (hpi). After RNA isolation and reverse transcription, the samples were analyzed via qPCR, using primers for 11 mRNAs of the Apobec/AID protein family, which were normalized to the housekeeping mRNA for TBP. The E1A mRNA levels, serving as infection controls, were measurable at 24 hpi and increased significantly at 48 hpi (Fig. 1A). We confirmed published results, showing moderate Apobec3B upregulation during HAdV infection (35). Apobec1 and Apobec4 were not detected. Apobec3C, D, E, and H were reduced at 48 hpi, whereas 3F, 3G, and AICDA (activation-induced cytidine deaminase) showed some virus-mediated changes, although they were not statistically significant. Most prominently, Apobec3A was strongly upregulated in parallel to HAdV E1A, with mRNA levels increasing up to 40-fold, compared to those observed at 0 hpi (Fig. 1A).

To confirm the upregulation of Apobec3A at the protein level, we infected human A549 cells with HAdV and checked endogenous Apobec3A protein levels at various time points postinfection via Western blotting. Apobec3A protein was clearly detected, starting from 48 hpi (Fig. 1B).

Although Apobec proteins are transcriptionally activated upon interferon (IFN) type I stimulation, Apobec3A transcription was so far shown to be firmly increased by the interferon treatment of myeloid lineage cells but not T cells (36–38). To investigate the IFN-mediated response of Apobec3A transcription in our experimental setup, A549 cells were treated with 1,000 U/mL IFN-α2b at 24 h prior to infection with mock or HAdV wt. The cells were harvested 48 hpi, and the IFN secretion was measured with the reporter cell line HL116 (Fig. 1C, left panel). mRNA was extracted and reverse transcribed, and a qPCR analysis for Apobec3A was performed. We found that HAdV increases Apobec3A transcription in untreated cells, which was not significantly altered in the HAdV-infected cells or the IFN-treated cells, as was the case for the mock IFN-treated cells versus the mock untreated cells (Fig. 1C).

To look more closely at the protein expression and potential protein stabilization, we used a more specific and easily detectable V5-tagged Apobec3A transfection system, in which Apobec3A expression is under the control of a CMV promoter, thus ruling out HAdV-mediated transcriptional activation. After transiently transfecting various cell lines with pLenti6.3-Apobec3A-V5, the exogenous Apobec3A protein levels were upregulated by HAdV infection in H1299, HeLa, HepaRG, HepaRG-His-SUMO2 (stably expressing 6×His tagged SUMO2), HepaRG-shCtrl (stably expressing scrambled shRNA), and HepaRG-shPML (stably expressing PML shRNA), and the signal intensities were normalized and calculated relative to β-actin (Fig. 1D). These findings indicated that in addition to increased gene expression (Fig. 1A–C), Apobec3A protein levels are also highly stabilized during HAdV infection (Fig. 1C and D).

We chose HepaRG cells for further experiments, based on our results above, which show the highest relative levels of Apobec3A mRNA and protein accumulation at 48 h post HAdV infection. Cells transfected with the pLenti6.3-Apobec3A-V5 expression vector and infected with HAdV were then treated with cycloheximide 48 h later, which induces a block in eukaryotic protein translation. The band intensities in the Western blots were calculated and normalized in the lower chart, thereby confirming Apobec3A protein stabilization during infection (Fig. 1E).

### Apobec3A protein is not destabilized by the E1B-55K/E4orf6 E3 ubiquitin ligase.

**FIG 2:**
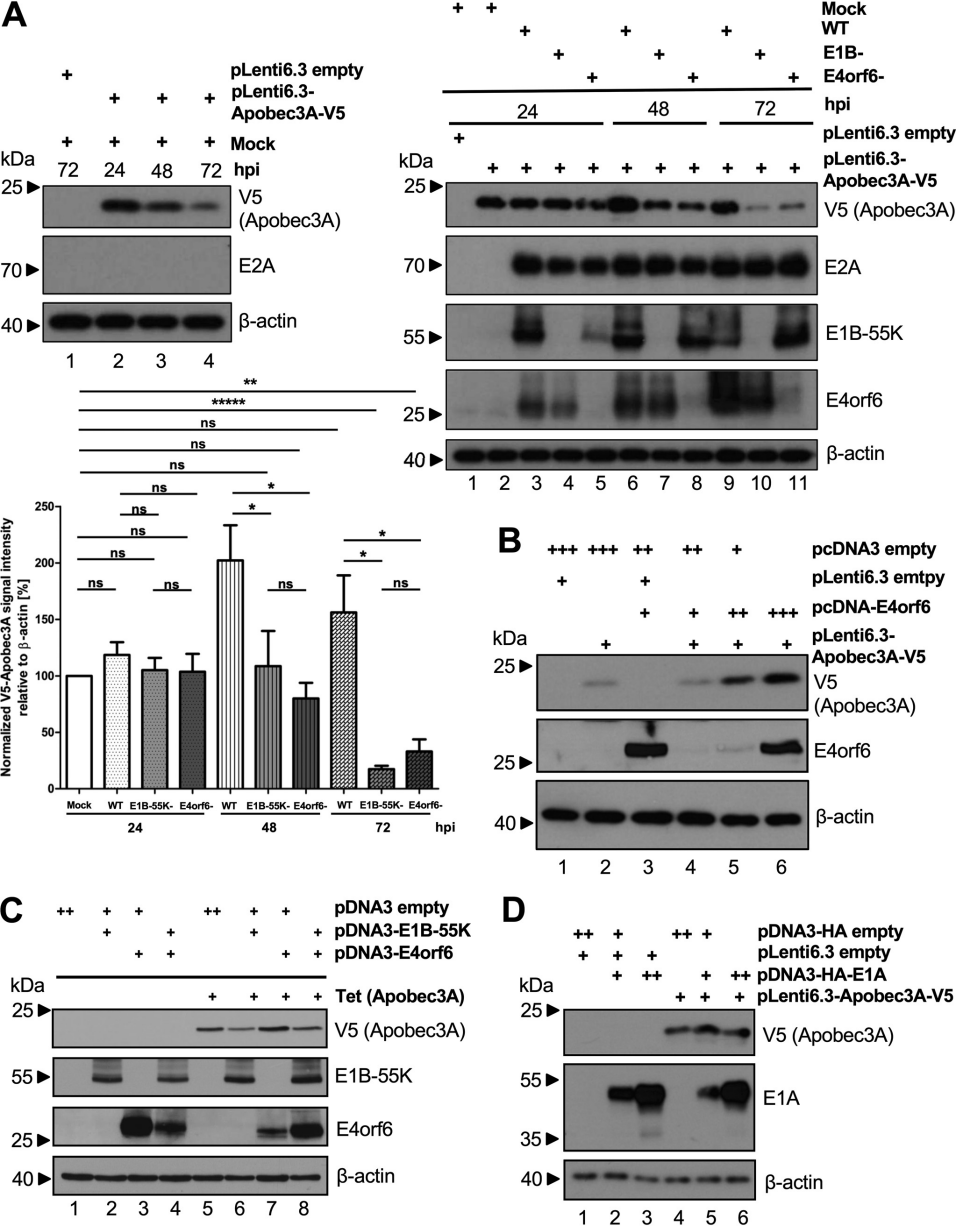
Apobec3A protein stabilization depends on both E1B-55K and E4orf6. Thus, it is not a substrate of the viral E1B-55K/E4orf6 E3 ubiquitin ligase complex. (A) HepaRG cells were transfected with 5 μg pLenti6.3-Apobec3A-V5, mock-infected, and harvested at 24, 48, and 72 hpi (left panel). Western blotting was performed as in Fig. 1. Also, HepaRG cells were transfected with 5 μg pLenti6.3-Apobec3A-V5 and mock-infected or infected with HAdV wt (50 ffu/cell), HAdV lacking functional E1B-55K (E1B-55K− (compare dash with E4orf6−)-; 50 ffu/cell), or HAdV lacking functional E4orf6 (E4orf6−; 50 ffu/cell). Cells were harvested at 24, 48, and 72 hpi and were analyzed via Western blotting as above, including E2A staining as an infection control (right panel). The signal intensities were calculated as in Fig. 1 (lower panel). (B) HepaRG cells were cotransfected with 5 μg pLenti6.3-Apobec3A-V5 and increasing amounts of pcDNA3-E4orf6 (5, 10, 15 μg; +, ++, +++, respectively). At 48 h after transfection, cells were harvested and analyzed via Western blotting as above. (C) HepaRG-TR-Apobec3A cells were induced with 5 μg/mL tetracycline (Tet). At 24 h post treatment, the cells were transfected with 5 μg empty pcDNA3 and pcDNA3-E1B-55K, or pcDNA3-E4orf6, or pcDNA3-E1B-55K and pcDNA3-E4orf6. Cells were harvested at 24 h posttransfection, whole-cell lysates were generated, and proteins were separated via SDS-PAGE and were subjected to Western blotting as above, including antibodies for E1B-55K, E4orf6, and V5 (Apobec3A), which served as a TR-induction control. (D) H1299 cells were transfected with 5 μg pLenti6.3-Apobec3A-V5 and 5 (+) or 15 μg (++) pcDNA3-E1A-HA and the corresponding empty vector, and they were harvested at 48 h posttransfection. Western blotting was performed as described above. Statistical analyses were performed using an unpaired *t* test (*, *P* < 0.05; **, *P* < 0.01; *****, *P < *0.0005; ns, not significant).

Since one of the key mechanisms used by HAdV for efficient replication is host protein ubiquitinylation by the E3 ubiquitin ligase complex, we looked more closely at the effect of E1B-55K and E4orf6 on the Apobec3A protein levels during HAdV infection. We first checked how stable transiently expressed Apobec3A protein is over time in the absence of HAdV. HepaRG cells transfected with pLenti6.3-Apobec3A-V5 or the corresponding empty vector were mock-infected and harvested at indicated time points. Apobec3A protein levels declined between 24 and 72 hpi (Fig. 2A, left panel). Next, these cells were then superinfected with either HAdV wt, E1B-55K minus or E4orf6 minus viruses. E2A served as a control for equal amounts of superinfected viruses; the detection of E1B-55K or E4orf6 confirmed the presence of the reciprocal virus mutant. As before, the wt virus maintained or even increased the Apobec3A protein levels that were detectable at 72 hpi and significant at 48 hpi. However, mutant viruses lacking functional E1B-55K or E4orf6 were not able to maintain Apobec3A protein levels to the same extent as was the wt virus (Fig. 2A, right panel), with the patterns at 48 and 72 hpi resembling that of the mock-infected time course control. The V5 Apobec3A signal intensities were calculated in a bar chart, confirming the significant upregulation at 48 and the continued stabilization at 72 h post wt infection (Fig. 2A, lower panel). This indicates that rather than destabilizing this cellular protein, both viral proteins are required, and the interplay between E1B-55K and E4orf6 together are necessary to increase Apobec3A levels during infection. The Western blot signal intensities were calculated from 3 independent replicates, and they confirmed the significant upregulation of Apobec3A at 48 h post wt infection, in comparison to the mock, as well as 72 hpi, in comparison to the minus virus infections (Fig. 2A, lower panel).

To investigate the possible role played by these two viral proteins in more detail, we first looked at the effect of E4orf6 alone on Apobec3A stabilization. We titrated increasing amounts of an E4orf6 expression vector together with steady-state cotransfection with pLenti6.3-Apobec3A-V5 and the samples being cotransfected with compensating amounts of empty vector to equalize the total transfection loads. Initially, the presence of Apobec3A hugely diminished the E4orf6 protein levels (Fig. 2B, lanes 3 and 4), but this was overcome by the levels of the E4orf6 expression vector increasing threefold. Interestingly, this correlated directly with the increasing Apobec3A levels, suggesting that the E4orf6 and Apobec3A proteins mutually affect each other and that in the absence of E1B-55K, the E4orf6 protein alone is destabilized by Apobec3A, but, when present in sufficient amounts, it can stabilize the Apobec3A protein (Fig. 2B, lane 6).

To analyze the effect of adding E1B-55K back into the above system and to reduce the transfection loads, we chose the HepaRG-TR-Apobec3A (V5-Apobec3A) cell line, in which the stable expression of Apobec3A can be induced by tetracycline (Tet). These cells, in either the absence or the presence of Tet, were transfected with E1B-55K as well as with E4orf6 expression vectors 24 h later (Fig. 2C). Again, the E4orf6 protein levels were severely diminished in the presence of Apobec3A (Fig. 2C, lanes 3 and 7), but they were rescued by the presence of E1B-55K (Fig. 2C, lanes 7 and 8).

To exclude the possible transactivation of the CMV immediate early promoter by the adenoviral transactivator E1A (39, 40), from which Apobec3A is expressed in the pLenti6.3 vector, we cotransfected 5 μg pLenti6.3-Apobec3A-V5 together with 5 (+) or 15 (++) μg pcDNA3-HA-E1A or the corresponding empty vector. Western blotting revealed that HAdV E1A does not alter CMV-driven Apobec3A expression during transfection (Fig. 2D, lanes 4 to 6), indicating that HAdV infection with functional E1B-55K and E4orf6 affects Apobec3A protein levels (Fig. 2A) and that the interplay between E1B-55K and E4orf6 stabilizes E4orf6, which in turn stabilizes Apobec3A.

### Apobec3A antagonizes HAdV gene expression and progeny production.

**FIG 3:**
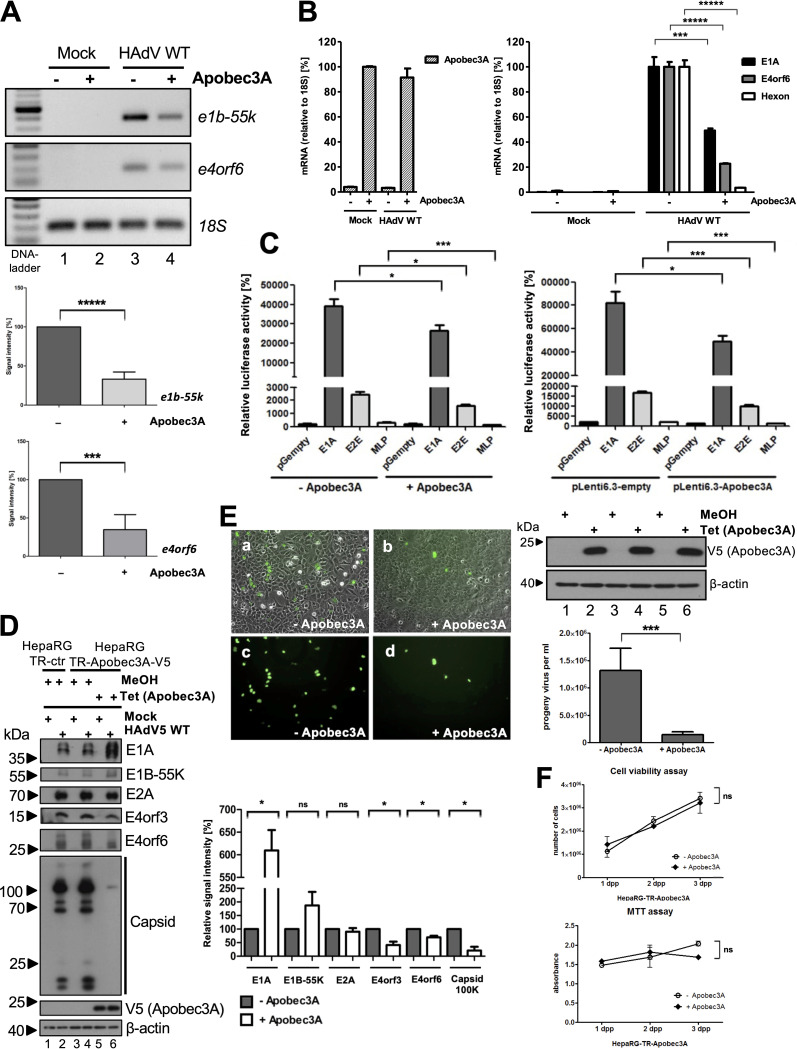
Apobec3A antagonizes HAdV DNA replication, gene expression, and progeny production. (A–E) HepaRG-TR-Apobec3A cells were induced with 5 μg/mL Tet for V5-tagged Apobec3A expression and were infected 24 h later with HAdV wt (50 ffu/cell). (A) Viral DNA synthesis was monitored by amplifying the *e1b-55* and *e4orf6* genomic region of HAdV via PCR. The PCR products were loaded on agarose gels. Human *18S* was used as a PCR control, and, to normalize the signal intensities of the bands on the agarose gel, calculated using the ImageJ program. (B) HepaRG-TR-Apobec3A-V5 cells were harvested at 24 hpi, mRNA was isolated, and cDNA was reverse transcribed. This was followed by qPCR analysis. The primers that were used to investigate Apobec3A mRNA were used as a Tet induction control. Primers for the early adenoviral mRNAs E1A and E4orf6 as well as for the late mRNA Hexon were used. 18S mRNA was used as a control, and signals were calculated in relation to it. (C) HepaRG-TR-Apobec3A-V5 cells were cotransfected with 0.5 μg renilla and 1 μg of the adenoviral promoters E1A, E2E, and MLP (left panel), or H1299 cells were cotransfected with 0.5 μg pLenti6.3-Apobec3A-V5, 0.5 μg renilla, and 1 μg of the same adenoviral promoters (right panel). At 24 h posttransfection, the cells were harvested, following a luciferase assay which was measured in a Tecan reader. (D) HepaRG-TR control and HepaRG-TR-Apobec3A cells were harvested at 48 hpi, whole-cell lysates were generated, and proteins were separated via SDS-PAGE and subjected to Western blotting as in Fig. 1, using antibodies against E1A, E1B-55K, E2A, E4orf6, and capsid, as well as V5 (Apobec3A) serving as a Tet-induction control, and β-actin as a loading control. The signal intensities were calculated using the ImageJ program (lower panel). (E) At 24 hpi, cells were harvested and resuspended in DMEM without supplements, and the virus was isolated via three freeze (liquid nitrogen) and thaw (37°C, water bath) cycles. HEK293 cells were reinfected with different virus dilutions and were fixed with methanol after 24 h. Immunostainings with E2A were performed, and viral progeny production was analyzed using an Axiovert 200 M microscope. Microscopic images (×10 magnification) show cells without Apobec3A expression (panels A and C) and cells with Apobec3A expression (panels B and D) with HAdV E2A stained in green (488 nm). An aliquot of the cells before reinfection was separated for the Western blot analysis to confirm the Tet-induction of Apobec3A. The antibodies V5 (Apobec3A) and β-actin were used as the induction and loading controls, respectively (lower left panel). The adenoviral progeny virus production was calculated by the mean of several counts of different visual fields, considering the dilution factor and microscope magnifications (lower right panel). (F) Cell viability and MTT assays were performed for the first 3 days, comparing Tet-induced (+ Apobec3A) versus noninduced (− Apobec3A) HepaRG-TR-Apobec3A cells (dpp, days post plating). All statistical analyses were performed using an unpaired *t* test (*, *P < *0.05; **, *P < *0.01; ***, *P < *0.005; ****, *P < *0.001; *****, *P < *0.0005; ns, not significant).

To investigate the function of Apobec3A during HAdV infection, we used the V5-Apobec3A inducible HepaRG-TR-Apobec3A cell line, in which the stable expression of V5-Apobec3A is induced with Tet, and we investigated key viral markers for productive infection. First, we studied the viral DNA levels via PCR, using primers for the genomic regions of *e1b-55k* and *e4orf6*. We observed that Apobec3A reduces HAdV DNA synthesis significantly during virus replication (Fig. 3A). Furthermore, a qPCR analysis of E1A, E4orf6, and Hexon mRNA showed that Apobec3A expression also strongly downregulates viral transcripts during infection (Fig. 3B).

To verify these data, we tested the effect of Apobec3A on HAdV promoters that were transfected in induced HepaRG-TR-Apobec3A cells (Fig. 3C, left panel) and in H1299 that was were cotransfected with either pLenti6.3 empty or pLenti6.3-Apobec3A-V5 (Fig. 3C, right panel). In induced HepaRG-TR-Apobec3A cells, Apobec3A expression repressed the activity of all of the viral promoters tested. E1A, E2E, and MLP consistently showed approximately 50% reduced promoter activity (Fig. 3C, left panel). In the transfected H1299 cells, the viral promoters E1A, E2E, and MLP also showed 40 to 50% reduced promoter activity (Fig. 3C, right panel).

To examine the effect on viral protein levels, we induced Apobec3A in HepaRG-TR-Apobec3A cells with Tet and infected them with HAdV. The HAdV E4orf3, E4orf6, and capsid protein levels were decreased significantly upon Apobec3A expression (Fig. 3D). E1A was significantly increased (Fig. 3D, lanes 4 and 6), possibly indicating a pressure on viral replication and an exaggerated transcriptional response. E2A and E1B-55K expression did not significantly change (Fig. 3D). We evaluated virus progeny production and found a significant decrease in the presence of Apobec3A expression (Fig. 3E). To exclude possible toxicity due to Tet and the induction of Apobec3A overexpression, we conducted cell viability and MTT assays. Comparing induced versus noninduced HepaRG-TR-Apobec3A cells (+/− Apobec3A) revealed no significant differences and led to the conclusion that Tet induction does not harm the cells under our culturing conditions in the applied time frame (Fig. 3F). To summarize these findings, we observed that Apobec3A is a novel restriction factor for HAdV gene expression, replication, and progeny virus production.

### Apobec3A transient knockdown promotes HAdV replication.

**FIG 4:**
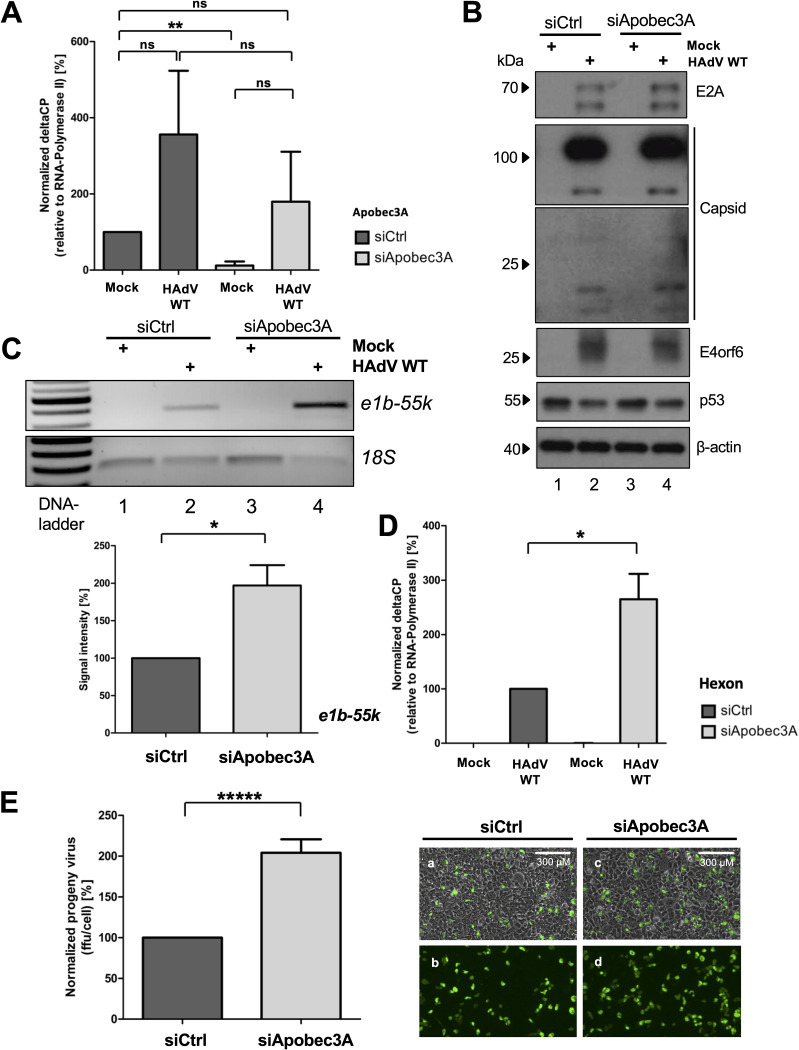
Apobec3A transient knockdown promotes HAdV replication. (A–E) HepaRG cells were transfected for 24 h with either siRNA Silencer Select Negative Control (siCtrl) or siApobec3A (200315) before infection with HAdV (50 ffu/cell). At 30 hpi, cells were harvested and subjected to analysis. (A) mRNA isolation, cDNA reverse transcription, and qPCR were performed from siRNA treated cells, as in Fig. 3. Apobec3A mRNA was set in relation to RNA-Polymerase-II. (B) Western blotting was done as in Fig. 1, using antibodies against E2A, capsid, and E4orf6 as infection controls, p53 as a HAdV degradation control, and β-actin as a loading control. (C) Viral DNA isolation, PCR, and agarose gel electrophoresis were performed as in Fig. 3. Band signal intensities were calculated using the ImageJ program. (D) mRNA isolation, cDNA reverse transcription, and qPCR were performed as in Fig. 3. Hexon mRNA was set in relation to RNA-Polymerase-II. (E) Adenoviral progeny virus production was performed, and images were taken of infected cells (right panel, subpanels a–d), as in Fig. 3. All statistical analyses were performed using an unpaired *t* test (*, *P < *0.05; **, *P < *0.01; *****, *P < *0.0005; ns, not significant).

To validate our previous data (Fig. 3), we transiently silenced endogenous Apobec3A and infected cells with HAdV wt at 24 h posttransfection. HepaRG cells were harvested at 30 hpi and were subjected to mRNA analyses and Western blotting. Additionally, virus progeny production was performed. As expected, the qPCR analysis of the control siRNA samples (siCtrl) showed increased levels of endogenous Apobec3A mRNA during HAdV infection, compared to the mock. Furthermore, we confirmed the transient knockdown of Apobec3A between the mock treated with the control siRNA and the siRNA against Apobec3A. Importantly, the calculation revealed that despite the siRNA treatment against Apobe3A, the HAdV infection chiefly overruled the knockdown of Apobec3A; however, the mean Apobec3A mRNA levels were decreased by approximately half, compared to the control siRNA during infection (Fig. 4A). The Western blot analysis served as the infection and loading control. The initial production of early (E2A, E4orf6) and late (capsid) proteins was not altered between transient silencing with the control or Apobec3A siRNA, nor was the degradation of p53 by HAdV (Fig. 4B, lanes 2 and 4). Nonetheless, the isolation and PCR amplification of the viral DNA revealed elevated HAdV DNA replication on the agarose gel and the corresponding calculation (Fig. 4C), and a qPCR analysis confirmed a significant rise in HAdV hexon mRNA during the silencing of Apobec3A (Fig. 4D). Lastly, the investigation of HAdV progeny production showed a significant increase in viral particles during transient Apobec3A knockdown, compared to the control (Fig. 4E). Taken together, as a proof-of-concept, we confirmed that the knockdown of Apobec3A elevates HAdV DNA, mRNA, and progeny, reaffirming our experiments with overexpression and strengthening the hypothesis of Apobec3A being an antiviral cellular factor against HAdV.

### Apobec3A interferes with the integrity of HAdV replication centers.

**FIG 5:**
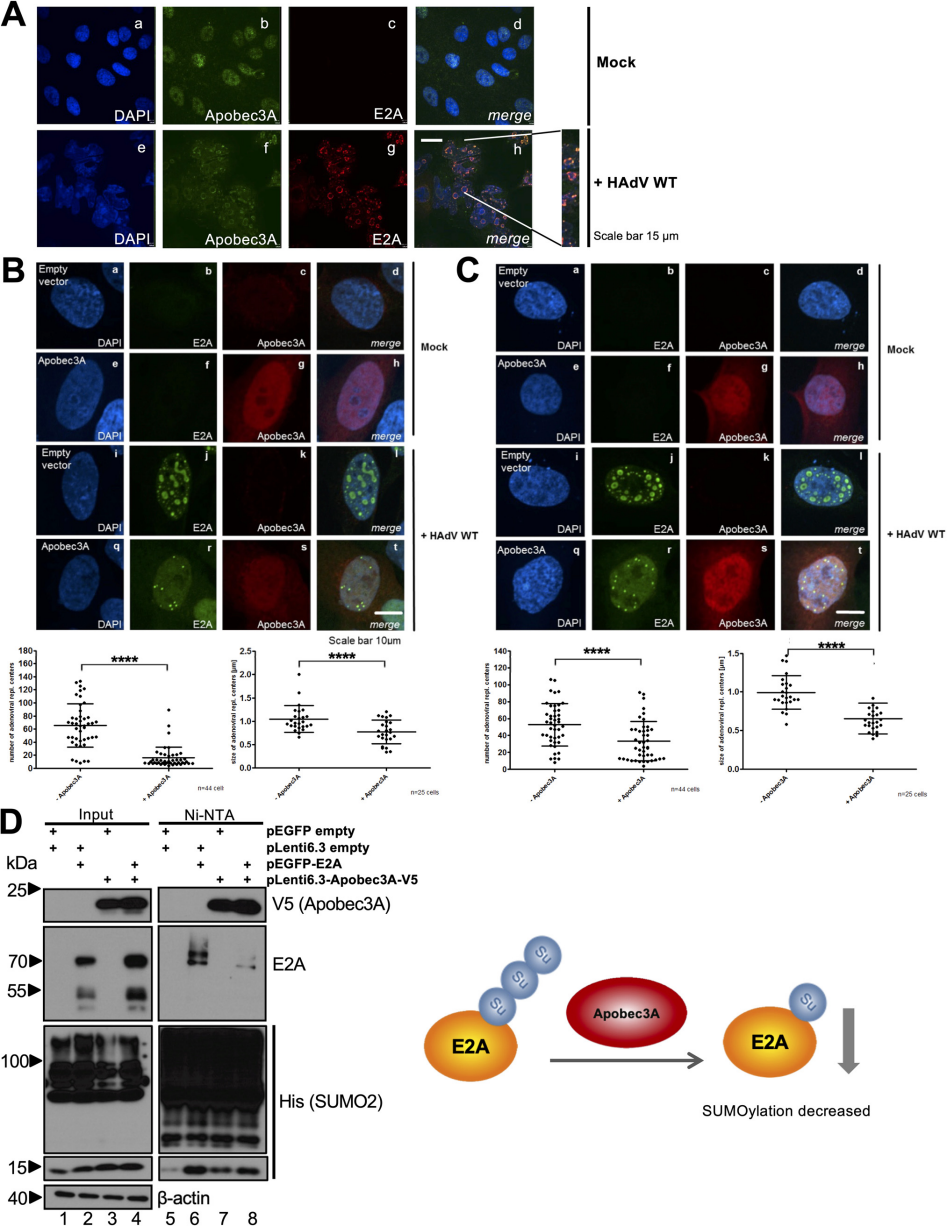
Apobec3A interferes with the integrity of HAdV replication centers. (A) A549 cells were mock-infected or infected with HAdV wt (20 ffu/cell) and harvested at 48 hpi. Fixed cells on coverslips were subjected to an immunofluorescence analysis, using nuclear DAPI staining, as shown in blue (405 nm), and antibodies for endogenous Apobec3A, as shown in green (488 nm), as well as E2A, as shown in red (647 nm). (B) HepaRG-TR-Apobec3A cells were either noninduced (subpanels a–d and i–l) or induced with 5 μg/mL Tet for Apobec3A expression (subpanels e–h) and infected with HAdV wt (50 ffu/cell; subpanels q–t). The cells were harvested at 24 hpi and subjected to an immunofluorescence analysis, using DAPI, as shown in blue (405 nm), and antibodies against V5 (Apobec3A), as shown in red (647 nm), as well as E2A, as shown in green (488 nm). The number and sizes of adenoviral replication centers were investigated (lower panel). (C) HepaRG cells were transfected with 5 μg pLenti6.3 empty vector (subpanels a–d and i–l) or with 5 μg pLenti6.3-Apobec3A-V5 (subpanels e–h) and infected with HAdV wt (50 ffu/cell; subpanels q–t). Cells were harvested at 24 hpi and subjected to immunofluorescence analysis, as in panel B. The number and sizes of adenoviral replication centers were determined, respectively (lower panel). (D) HeLa-HisSUMO2 cells stably expressing His-SUMO2 were transfected with 5 μg of pLenti6.3 empty, pLenti6.3-Apobec3A-V5, and/or pEYFP empty and pEGFP-E2A. At 24 hpi, the cells were harvested, and a Ni-NTA assay as well as cell lysates were performed. Proteins were separated via SDS-PAGE and were subjected to Western blotting, as in Fig. 1, using antibodies against V5 (Apobec3A), GFP (E2A), 6xHis (SUMO2), and β-actin as a loading control. (E) Schematic representation of Apobec3A influence on E2A SUMO modification. Statistical significance was assessed using an unpaired *t* test (****, *P < *0.001).

To investigate the localization of Apobec3A during HAdV infection, we performed immunofluorescence studies and monitored endogenous host protein expression. We found Apobec3A localizing in E2A-containing replication centers throughout the whole nucleus in infected cells (Fig. 5A, subpanel h). Using the more easily detectable Apobec3A-V5 constructs to validate the restrictive function of Apobec3A on HAdV replication, we compared the replication centers in HAdV-infected HepaRG-TR-Apobec3A cells that had been mock-induced or induced with Tet (+ Apobec3A). Interestingly, replication centers, as marked by the early viral protein E2A, were decreased in number and size upon Apobec3A overexpression (Fig. 5B, subpanel t). To statistically validate this effect, we counted the replication centers and measured their sizes in different cells, revealing a significant reduction in the presence of Apobec3A (Fig. 5B, lower panel). To confirm our results, we performed a similar experiment in HepaRG cells that were transfected with pLenti6.3-Apobec3A-V5 and were infected with HAdV, and we observed the same effects (Fig. 5C, subpanel t). Thus, we consistently observed that Apobec3A reduced E2A-containing viral replication centers in number and size (Fig. 5B and C, lower panels).

Recent data from our group indicates that E2A SUMO modification is essential for efficient infection, which shows higher migrating bands in NiNTA blots, representing SUMO chains on the protein of interest (41, 42). Thus, we next tested whether Apobec3A modulates E2A SUMOylation, possibly impairing HAdV replication centers. HeLa-His-SUMO2 cells, stably expressing 6×His tagged SUMO2, were cotransfected with pLenti6.3-Apobec3A-V5 and E2A-GFP. Ni-NTA assays, which pull down His-SUMO2 and His-SUMOylated proteins, showed that Apobec3A expression significantly decreases E2A SUMOylation (Fig. 5D, lanes 6 and 8). Since E2A SUMOylation is necessary for correct HAdV replication center formation, Apobec3A may impair these by decreasing E2A SUMOylation.

### Apobec3A selectively deaminates HAdV DNA sequences.

The deaminase activity of Apobec3A is required for the restriction of many viruses. Therefore, we next investigated any Apobec3A deaminase activity during HAdV infection by performing a differential DNA denaturation PCR (3D-PCR) (43). Upon cytosine conversion, uracil-N-glycosylases (UNG) excise mismatched uracils from the DNA and initiate the base-excision repair (BER) pathway, which patches lesions within dsDNA (44, 45). UNG inhibitors (UGI) stabilize deamination processes (46). What one might then expect to observe is that the opposite strand DNA sequence contains G to A conversions. Experimentally, deamination results in DNA PCR amplicons that contain a larger amount of A-U(T) base pairs with only two hydrogen bonds. Thus, they denature at lower temperatures. Consequently, bands emerging at lower denaturing temperatures than in the controls indicate deamination processes.

**FIG 6:**
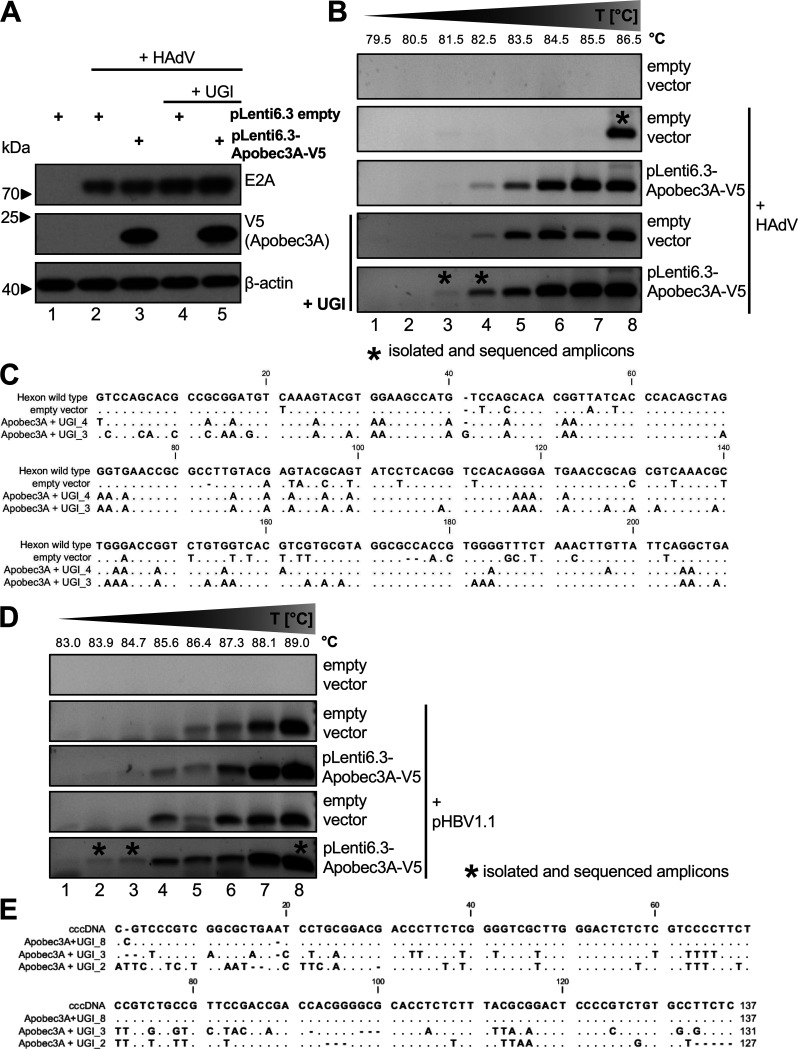
Apobec3A deaminates HAdV DNA sequences. (A) HepaRG-TR-UGI cells were either noninduced or induced with 5 μg/mL Tet to express UGI and were transfected with 5 μg pLenti6.3-Apobec3A-V5, which was followed by infection with HAdV wt (50 ffu/cell). At 72 hpi, the cells were harvested, and an aliquot was subjected to Western blotting, as in Fig. 1, using antibodies against E2A as an infection control as well as V5 (Apobec3A) and β-actin as a loading control. (B) The samples harvested in panel A were subjected to a 3D-PCR analysis, using primers for a CT-rich region in the Hexon amplicon as well as denaturing temperatures that ranged from 79.5 to 86.5°C. 3D-PCR results were analyzed on a 1% agarose gel. (C) The indicated amplicons (*) in panel B were isolated, sequenced, aligned, and subsequently compared to the wt sequence of Hexon using CLC Workbench (Qiagen). (D) HepaRG-TR-UGI cells were either noninduced or induced with 5 μg/mL Tet for UGI expression and were cotransfected with 5 μg pLenti6.3-Apobec3A-V5 as well as 10 μg of the pHBV1.1 plasmid, which establishes HBV infection. At 72 hpi, the cells were harvested using a NucleoSpin Tissue Kit (Machery Nagel) and were subjected to a 3D-PCR analysis, using cccDNA primers as well as different denaturing temperatures that ranged from 83 to 89°C. The 3D-PCR results were analyzed on a 1% agarose gel, similar to that described in panel B. (E) The indicated amplicons (*) in panel D were isolated, sequenced, aligned, and subsequently compared to the wt sequence of HBV cccDNA using CLC Workbench.

HepaRG-TR-UGI cells, in which UGI expression is induced with Tet, were transfected with pLenti6.3-Apobec3A-V5 and were superinfected with HAdV (Fig. 6A). At 72 hpi, the cells were harvested, and the isolated viral DNA was subjected to a 3D-PCR analysis of the adenoviral hexon amplicon (Fig. 6B). For the HAdV-infected samples, both Apobec3A overexpression in the absence of UGI and empty vector transfection in the presence of UGI yielded hexon amplicons at lower denaturing temperatures, suggesting that in the latter, endogenous deamination is stabilized when UGI prevents UNG-induced DNA repair (Fig. 6B). Notably, the HAdV-infected samples that were transfected with Apobec3A-V5 and induced for UGI expression generated a band at even lower denaturing temperatures, which indicated even higher levels of stabilized deamination in the hexon amplicon, mediated by exogenous Apobec3A (Fig. 6B, bottom panel). The amplicons marked with an asterisk (Fig. 6B, lanes 3, 4, and 8) were isolated and sequenced, and the results were aligned and compared with the hexon wt sequence. We found various G to A transitions, indicating the Apobec3A-mediated deamination of C nucleotides in the virus minus-strand (Fig. 6C). Interestingly, and as might be expected, the amplicon with the lowest denaturing temperature (UGI_3) contained the most base pair substitutions.

As a control for the 3D-PCR assay, we included HBV, as a known Apobec3A deamination target amplicon, into our assays (27). HepaRG-TR-UGI cells induced with Tet were cotransfected with pLenti6.3-Apobec3A-V5 and the pHBV1.1 plasmid encoding a replication-competent HBV genome. 3D-PCR was performed on isolated cccDNA and confirmed HBV-DNA deamination by Apobec3A (Fig. 6D). Empty vector transfection with UGI induction also yielded cccDNA amplicons at lower denaturing temperatures, similar to those observed under Apobec3A overexpression, suggesting possible endogenous deamination as described above (Fig. 6A, lanes 3 and 4). The isolation and sequencing of the indicated amplicons (Fig. 6D, lanes 2, 3, and 8), followed by the alignment and comparison to the HBV wt sequence, confirmed mostly C to T transitions and some G to A transitions (Fig. 6E), reflecting the more complex replication steps that involve the reverse transcription of the pregenomic RNA (47).

### HAdV species A, C, and F evolution may have resulted in the depletion of TC dinucleotides.

**FIG 7:**
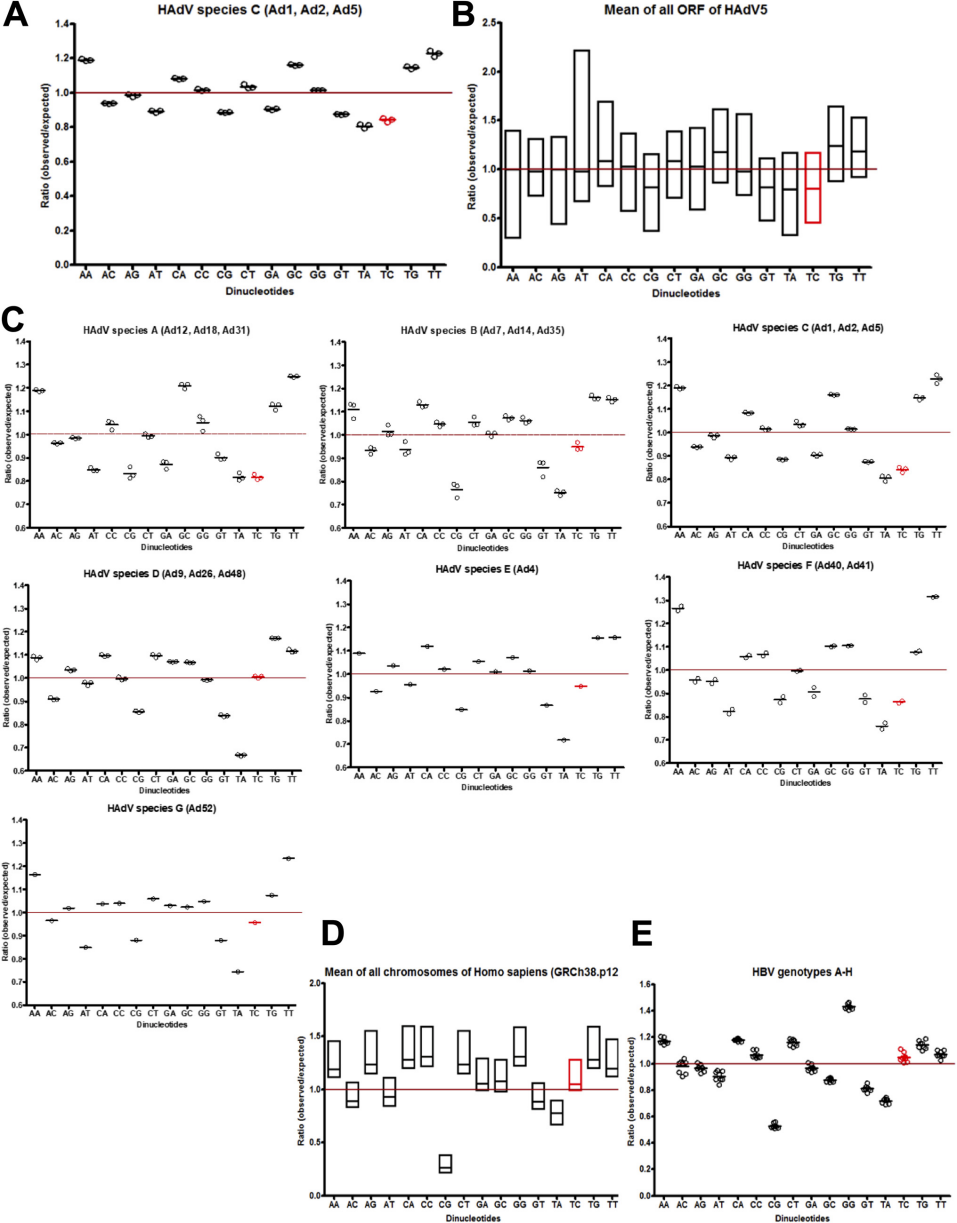
Dinucleotide frequencies in viral and human sequences. (A–E) The online tool compseq (Emboss) was used to determine the dinucleotide frequencies. The program calculates the observed versus the expected frequencies of dinucleotides in a given sequence, considering the amount of A, T, C, and G nucleotides in the sequence. The sequences were obtained from the nucleotide database of NCBI. The red line indicates where the observed frequency equals the expected frequency. The results for the TC dinucleotide, the main minimal target of Apobec3A, are marked in red. (A) The genomes of HAdV species C types 1, 2, and 5 were analyzed. (B) The mean of all ORFs of HAdV type C5 were investigated for their dinucleotide frequencies. (C) The dinucleotide frequencies of the members of the HAdV species A, B, C, D, E, F, and G were investigated. (D) The mean of all chromosomes of the reference genome for Homo sapiens (GRCh38.p12) was studied for the dinucleotide distributions. (E) The HBV genome was investigated for its dinucleotide distributions.

Apobec3A preferentially deaminates cytosines within the TpC context, although further factors such as the DNA secondary structure or the sequence context influence the deamination efficiency (48–50). Since HAdV of species C type 5 is restricted by Apobec3A, we hypothesized that also other HAdV types might have evolved to escape Apobec3A deamination. The online tool compseq (Emboss) was used to investigate the frequencies of the different dinucleotides within the adenoviral genome. The observed versus the expected dinucleotide frequency within the given sequence was calculated, while considering the amounts of A, T, C, and G nucleotides in the sequence. Investigating the genomes of the HAdV species C types 1, 2, and 5 led to the observation that the TC dinucleotides were approximately 20% less frequent than expected (Fig. 7A). Furthermore, TC dinucleotides are depleted in the open reading frames (ORFs) of HAdV species C type 5 (Fig. 7B).

We also determined the dinucleotide frequencies in the other HAdV species A, B, D, E, F, and G. TC depletion was only observed for the HAdV species A, C, and F (Fig. 7C). We did not detect TC depletion in the reference genome for Homo sapiens (GRCh38.p12; Fig. 7D) or in the HBV genome (Fig. 7E), indicating that, along with the HPV genomes (51), TC dinucleotide underrepresentation may be specific for the HAdV species A, C, and F types, but it does not represent a general or conserved mechanism among viruses. Of note was the consistent underrepresentation of CG and TA dinucleotides (as well as GT in the HAdV species) in Homo sapiens and in the HBV genomes, again suggesting that further evolutionary factors are conserved across biological domains (Fig. 7A–E).

### HAdV infection does not significantly alter Apobec3A SUMOylation.

**FIG 8:**
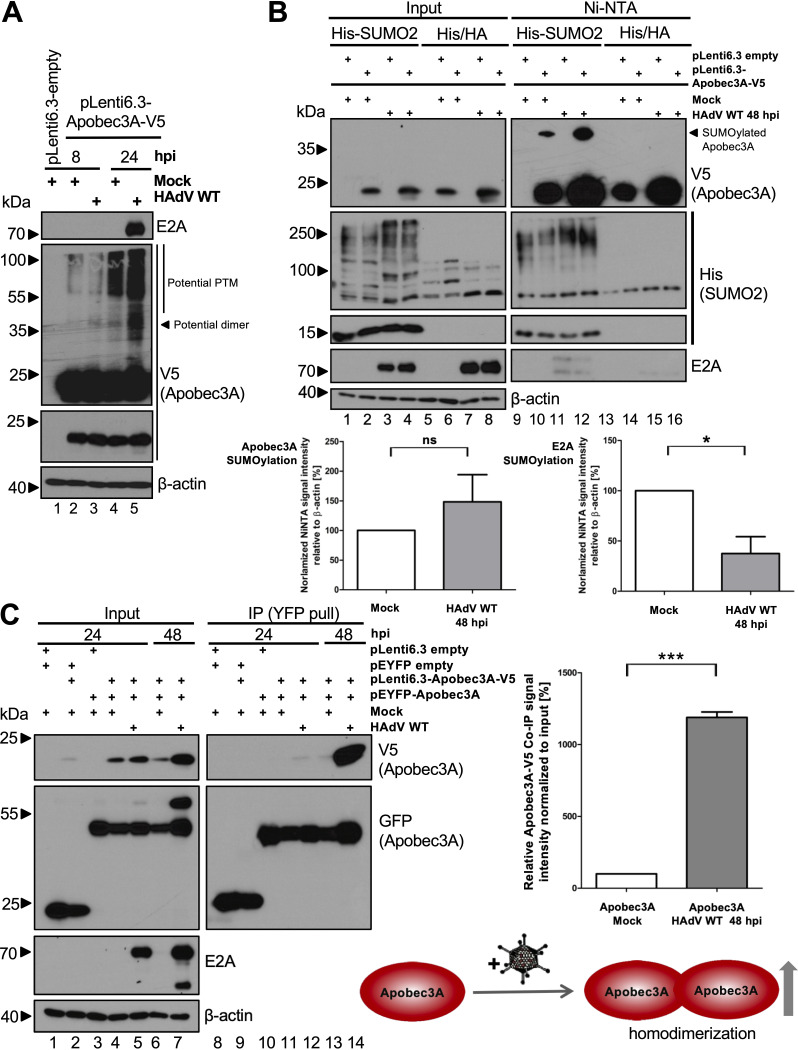
The determination of Apobec3A posttranslational modifications and Apobec3A dimer formation during infection. (A) HepaRG cells were transfected with 5 μg pLenti6.3 empty vector or with 5 μg pLenti6.3-Apobec3A-V5 and were infected with HAdV wt (50 ffu/cell). At 8 and 24 hpi, the cells were harvested, and whole-cell lysates were performed. One aliquot of the lysate, prepared without the addition of β-mercaptoethanol and boiling at 95°C, was subjected to Western blotting, as in Fig. 1, to detect Apobec3A with V5 antibody staining. E2A served as an infection control, and β-actin was used as a loading control. (B) HepaRG-His and HepaRG-His-SUMO2 cells were transfected and infected as in panel A. At 48 hpi, the cells were harvested, and whole-cell lysates as well as a Ni-NTA assay were carried out. The proteins were separated via SDS-PAGE and were subjected to Western blotting, as in Fig. 1, using antibodies against V5 (Apobec3A) and 6xHis (SUMO2) as cellular controls, E2A as an infection control, and β-actin as a loading control. The ImageJ program was used to calculate signal intensities. Signal intensities of slower migrating V5 bands in the NiNTA pulldowns were normalized to the Apobec3A V5 input bands, and the Apobec3A input levels were normalized on the β-actin levels. (C) HepaRG cells were transfected with either 5 μg pLenti6.3 empty vector or 5 μg pLenti6.3-Apobec3A-V5 and/or pEYFP empty vector or pEYFP-Apobec3A, and they were infected with HAdV wt (50 ffu/cell). Following harvesting at 24 hpi and 48 hpi, whole-cell lysates as well as coimmunoprecipitation using ChromoTek GFP-Trap agarose beads were performed. The proteins were separated via SDS-PAGE and were subjected to Western blot immunostaining, as in Fig. 1, using the antibodies against V5 (Apobec3A), GFP (Apobec3A), E2A as infection controls, as well as β-actin as a loading control. The ImageJ program was used to determine the signal intensities, and the signals for the V5-Apobec3A coimmunoprecipitation were normalized to the V5-Apobec3A input levels, relative to β-actin. The lower panel shows a schematic representation of Apobec3A homodimer formation during infection. Statistical analyses were performed on the calculations using an unpaired *t* test (*, *P* < 0.05; ***, *P < *0.005; ns, not significant).

Although Apobec3A deaminates HAdV amplicons and restricts the HAdV gene expression of the investigated E4 region and the capsid, the virus apparently does not target Apobec3A for the proteasomal degradation via the E1B-55K/E4orf6 E3 ubiquitin ligase to eliminate this novel host restriction factor (Fig. 2A). However, HAdV also potently makes use of host PTM machineries, especially SUMOylation (42, 52–55). Thus, we evaluated whether the virus-mediated modulation of Apobec3A PTM is a viral strategy by which to counteract Apobec3A. To visualize potential PTMs of Apobec3A, HepaRG cells were transfected with pLenti6.3-Apobec3A-V5 and superinfected with HAdV wt (Fig. 8A). Samples that were prepared under nondenaturing and nonreducing conditions were used to detect V5-tagged Apobec3A in a Western blot analysis, revealing an increase in Apobec3A PTM or oligomeric structures upon HAdV infection (Fig. 8A, lanes 4 and 5).

To investigate whether Apobec3A SUMOylation may be increased during infection, HepaRG-His-SUMO2, stably expressing 6×His-SUMO2, and control HepaRG-His/HA cells were transfected with pLenti6.3-Apobec3A-V5 and were superinfected with HAdV wt. Ni-NTA assays indicated that the SUMOylation of Apobec3A was only detected in HepaRG-His-SUMO2 cells and was not detected in the control cell line (Fig. 8B, lanes 10 and 14). Furthermore, Apobec3A SUMOylation was slightly increased upon HAdV infection; however, this result was not statistically significant (Fig. 8B, lanes 10 and 12), as determined by the normalization of the slower migrating band of the Apobec3A V5 NiNTA pulldown signals, relative to the V5 input levels (Fig. 7B, right panel). However, confirming earlier data (Fig. 5D), E2A SUMOylation was significantly decreased upon Apobec3A expression (Fig. 8B, lanes 11 and 12).

### HAdV infection significantly promotes Apobec3A dimer formation.

Apobec3A can form homodimers, which catalyze deamination efficiently (31). To investigate the Apobec3A dimer formation during HAdV infection, pLenti6.3-Apobec3A-V5 was either coexpressed with the pEYFP empty vector or with pEYFP-Apobec3A, and, *vice versa*, pEYFP-Apobec3A with the pLenti6.3-empty-V5 vector or pLenti6.3-Apobec3A-V5. The YFP empty vector controls were detected using the GFP antibody in the Western blot analysis as bands running below 23 kDa (Fig. 8F, lanes 1, 2, 8, and 9), whereas slower migrating bands above 55 kDa may represent YFP Apobec3A dimers. Pulling down the YFP tag of Apobec3A resulted in the detection of a weak signal for V5-tagged Apobec3A in the mock, which increased during HAdV infection, particularly at 48 hpi (Fig. 8F, lanes 13 and 14). The V5 signal intensities were normalized on the input levels and confirmed an approximately 10-fold increase in the homodimerization of Apobec3A at 48 h after HAdV infection, compared to the mock controls (Fig. 8F, right panel). In conclusion, HAdV infection induces Apobec3A dimer formation (Fig. 8F, lower panel), which may increase Apobec3A activity and/or protein stabilization to support the cell-intrinsic antiviral defense.

## DISCUSSION

Here, we show that overexpressed Apobec3A counteracts HAdV infection, thereby decreasing HAdV DNA replication, viral promoter activity, mRNA and protein levels, virus progeny production, and HAdV replication centers (Fig. 3). In accordance with overexpression, the siRNA mediated knockdown of Apobec3A increased HAdV DNA replication, mRNA levels, and progeny (Fig. 4). Moreover, during HAdV infection, Apobec3A protein levels increase due to changes at both the transcriptional and protein level due to the endogenous Apobec3A mRNA being increased (Fig. 1A) as well as CMV-driven Apobec3A expression being stabilized (Fig. 2B), even in the presence of cycloheximide (Fig. 1E). IFN did not significantly alter Apobec3A transcription in A549 cells, indicating that the HAdV-mediated Apobec3A response is independent of IFN, at least in our experimental setup (Fig. 1C). As A549 are lung-derived and represent HAdV-C5 target cells, we surmised this to be an adequate control for a natural HAdV-C5 infection within the context of IFN. Our data suggest that IFN may not play a central role in Apobec3A protection against HAdV in these tissues. Of course, different cell lineages might yield different responses of Apobec transcription (36–38).

Our infection studies with HAdV mutant viruses show that the E1B-55K/E4orf6 E3 ubiquitin ligase does not target Apobec3A for proteasomal degradation to eliminate this novel host restriction factor (Fig. 2A). In fact, the presence of E1B-55K and E4orf6 are required to stabilize Apobec3A. In the absence of E1B-55K, Apobec3A expression severely depleted the E4orf6 protein, which, when boosted by increasing the expression of E4orf6, led to increasing levels of Apobec3A. Thus, E1B-55K seems to be required to stabilize E4orf6, which in turn plays a critical role in enabling E4orf6 to stabilize Apobec3A.

Apobec3A protein stabilization may depend on increased homodimerization during HAdV infection. Recently, Bohn and colleagues found that Apobec3A forms homodimers via a symmetric swap of its N-terminal residues, which is believed to regulate Apobec3A activity (31). The homodimer interface connects the active sites of both monomers and thereby forms a positively charged groove, which is important for substrate recognition and specificity (31). Thus, stabilized homodimers possibly represent a novel cellular antiviral defense strategy to ensure adequate amounts of Apobec3A to counteract a HAdV infection. Indeed, our immunoprecipitation experiments indicated an increase in Apobec3A dimers during adenoviral infection (Fig. 8C). Nevertheless, the exact cellular mechanisms of Apobec3A protein regulation during HAdV infection still need to be elucidated.

Intriguingly, Apobec3A can deaminate HAdV DNA, which may be one of the key mechanisms by which Apobe3A restricts HAdV (Fig. 6A–C). Since uracil-N-glycosylases excise mismatched uracils from DNA, they initiate BER and DNA breaks, thereby activating DDR (18). However, HAdV must suppress DDR to replicate efficiently (34), suggesting that Apobec3A exerts replicative pressure on HAdV. Apobec3A overexpression also reduced E2A SUMOylation (Fig. 5), possibly impairing the formation of adenoviral replication centers, which indicates an additional and novel mechanism of the host-mediated interference of efficient HAdV replication. One might speculate that the silencing of Apobec3A may promote or even increase RC formation and, possibly, the SUMOylation-dependent E2A assembly of RC. However, this remains to be investigated.

Apobec3A restricts a variety of viruses, including HIV, HPV, HBV, HTLV, and parvoviruses (25–29), and this depends on its deaminase activity in most cases, except for parvoviruses, which are inhibited by a yet unknown, deaminase-independent mechanism (25–29). To study deamination, we investigated a region in the Hexon amplicon that contained many TC dinucleotides, the main minimal motif deaminated by Apobec3A (Fig. 6A–C) (36, 48–50). The subsequent isolation and sequencing of several amplification products of the 3D-PCR revealed many G to A transitions, indicating the preferential minus-strand deamination of the adenoviral Hexon amplicon. Minus-strand specificity was already observed for the Apobec3G editing of the HIV genome (56), and Apobec3A and Apobec3B preferentially deaminate ssDNA from the lagging strand during DNA synthesis (57). Thus, preferential minus-strand deamination might be a conserved mechanism for the Apobec restriction of different viruses. Furthermore, we observed the Apobec3A editing of the HBV cccDNA with a large amount of C to T mutations and some G to A mutations (Fig. 6D and E) (27, 28), which reflects the more complex replication steps that involve the reverse transcription of the pregenomic RNA to form the cccDNA (47).

In the present work, the genomes of HAdV species C were found to have less frequent TC dinucleotides in the genome (Fig. 7) (48, 50). This is in line with the HPV evasion of Apobec3A, as HPV has also evolved toward TC depletion (51). For HAdV A, C, and F, as well as the HPV genomes, the observed versus expected ratio of TC dinucleotides was reduced to 70 to 80% in the respective sequence (51). However, TC depletion neither represented a general mechanism nor was conserved among different viruses, as TCs were represented to an expected amount in the reference genome for Homo sapiens as well as the in HBV genome and in the different HAdV species (B, D, E, and G). Our investigations suggest that HAdV C species may evade Apobec3A deamination by less abundant TC dinucleotides. Yet, it would be interesting to investigate Apobec3A deamination and antiviral capacity on other HAdV species with expected TC frequencies. It is noteworthy that TAs (stop codon and TATA box) and CGs (methylation hot spots) are almost frequently underrepresented, whereas GCs can occur more frequently (methylation-deamination conversion) (58–60), which also seems to translate to most HAdV genomes (Fig. 6). Nevertheless, dinucleotide frequencies are associated with certain specific genomic events, whereas further mechanisms are still unaccounted for. Thus, we hypothesize that Apobec3A may potentially exert certain evolutionary pressures on some HAdV types but that other genomic pressures must also exist.

We observed that Apobec3A was SUMOylated, even in the absence of HAdV, which was increased after viral infection to a certain extent, although this result was not statistically significant under our experimental setup (Fig. 8B). However, this also deserves future investigation, since some of the residues that are predicted to be SUMOylated by the GPS SUMO (The CUCKOO Workgroup) program are known to be crucial for the dimer interface of Apobec3A (31). Among other residues, lysine 30 and 60 bridge the dimer interface and contribute to the positive charge and shape of the putative DNA-binding groove, thereby affecting substrate affinity and deamination (31). K30 and K60 are conserved among Apobec3A orthologues of different primates, emphasizing their crucial roles for Apobec3A functions (61). However, despite the performance of an Apobec3A mutational analysis, it is still unclear how many and which lysine residues are SUMOylated in the Apobec3A sequence. Prospective studies will be required to obtain an Apobec3A mutant for analyzing Apobec3A SUMOylation. We hypothesize that upregulated Apobec3A SUMOylation is required for the increased formation of Apobec3A homodimers during adenoviral infection, representing an antiviral defense of the host cell. However, since it was demonstrated that an increase in Apobec3A expression or protein stabilization is not conserved among different viruses (25–29), it would be interesting to examine whether the upregulation of Apobec3A dimer formation is specific for HAdV or is conserved among different viruses.

**FIG 9:**
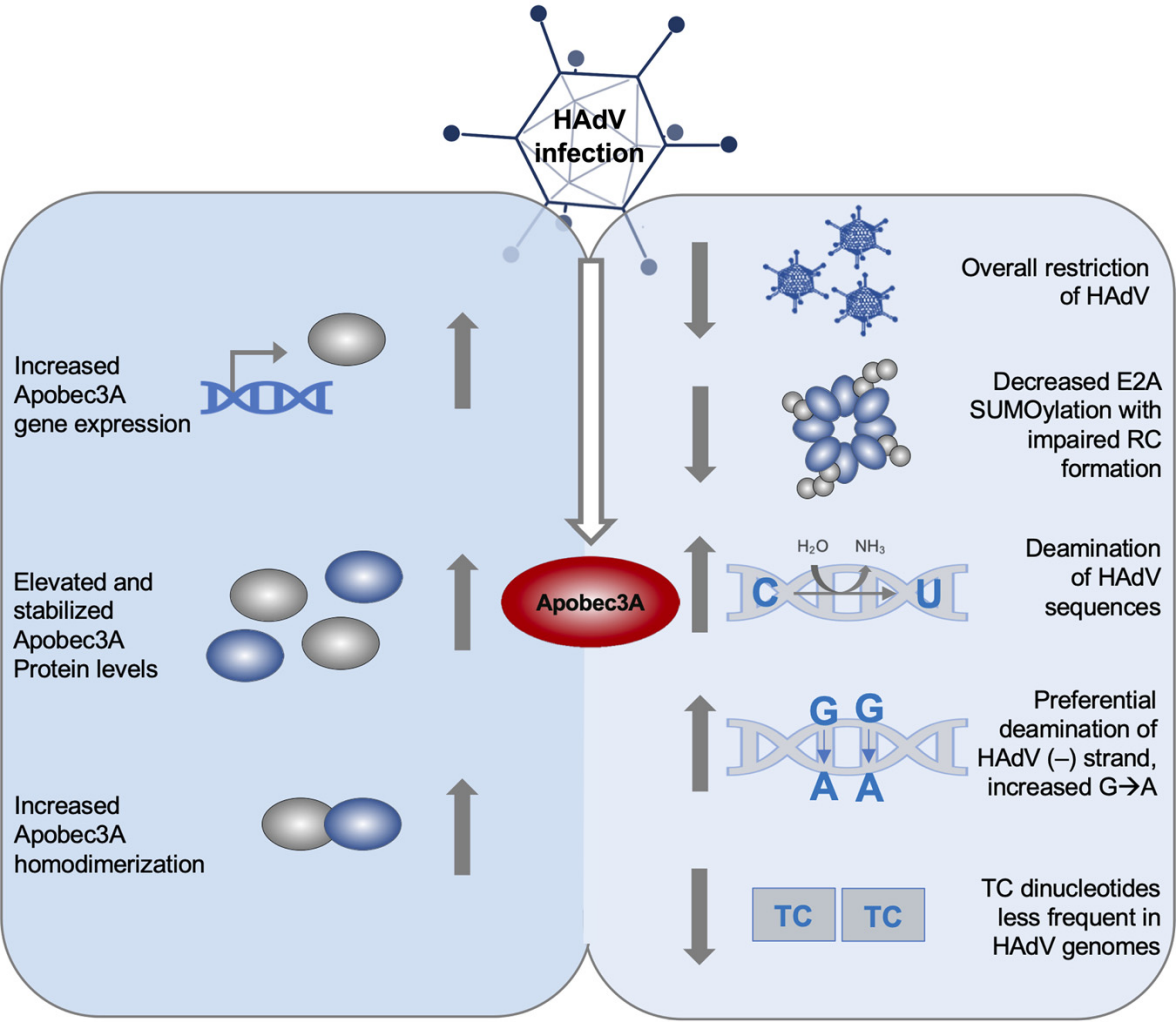
Role of Apobec3A during HAdV infection. Schematic representation of the observed findings in the present study. HAdV infection increases Apobec3A expression levels, protein stabilization, and its dimer formation, which represents an antiviral response of the host cell. Apobec3A inhibits HAdV infection through decreasing E2A SUMOylation and deaminating HAdV DNA, preferentially on the minus strand, although the virus possibly counteracts Apobec3A restrictive functions by featuring reduced TC dinucleotide frequencies.

In this study, we mainly focused on protein-protein interactions and PTMs with an exogenously expressed, V5-tagged Apobec3A. Future studies will be required to investigate the transcriptional regulation of endogenous Apobec3A. In conclusion, we found that Apobec3A is a novel restriction factor for HAdV infection (Fig. 9). Once the mechanisms are elucidated in detail, this will help provide insights into early host-virus interactions and viral oncogenesis. Such knowledge could improve current therapeutic strategies for the treatment of HAdV infections as well as vector applications of HAdV in the fields of gene therapy and vaccination.

## MATERIALS AND METHODS

### Cell culture.

Human lung carcinoma H1299 (ATCC Global Bioresource Center, No. CRL-5803), HEK293 (ECACC European Collection of Authenticated Cell Cultures; Sigma-Aldrich, No. 85120602-1VL), A549 (ECACC; Sigma-Aldrich No. 86012804), HeLa (ECACC; Sigma-Aldrich, No. 93021013), HeLa-His-SUMO2 (62), and HL116 (63) cells were cultured in Dulbecco’s modified Eagle’s medium supplemented with 10% fetal calf serum, 100 U of penicillin, and 100 μg of streptomycin. For HepaRG parental (Sigma-Aldrich, No. MTOX1010) and HepaRG-derived cells (HepaRG-shCtrl, HepaRG-shPML, HepaR-His-SUMO2, -His/HA, -TR, TR-UGI, and -TR-Apobec3A), the media were supplemented with 0.5 μM hydrocortisone and 5 μg/mL bovine insulin. All of the cell lines were cultured in 5% CO_2_ at 37°C.

Tetracycline (TR/Tet)-inducible HepaRG-TR-Apobec3A-V5 and HepaRG-TR control cells were generated as described previously (64), with a 5′ TR-regulated minimal CMV-promoter and a 3′ V5 tag. Inducible TR-Apobec3A-V5 or the corresponding TR control cells were selected with 10 μg/mL Blasticidin and 300 μg/mL Zeocin (64, 65). HepaRG-TR-UGI cells were established with the plasmid pLenti4-TO-UGI from human uracil-N-glycosylase (UNG) (primers UGI fwd, rev TTTGAATTCACCATGACCAACCTGAGC, TTCCTCGAGTCACAGCA TCTTGATCTTG; kindly provided by Michael Emerman/University of WA, USA) (66). V5-tagged Apobec3A or TR-UGI expression was either induced with 5 μg/mL tetracycline dissolved in methanol (+Tet) or mock-induced with methanol (−Tet) at 24 h prior to the experimentation. The corresponding control TR-cells were equally induced. HepaRG-His/HA-SUMO2 cells stably expressed 6×His-SUMO2 and control cells HepaRG-His/HA stably expressed 6×His/HA (kindly provided by Ron Hay/University of Dundee, UK) (67) were selected with 2 μM puromycin. All of the cell lines were regularly tested for the absence of mycoplasma.

### Plasmids and transient transfections.

Subconfluent cells were transiently transfected with PEI (polyethylenimine) and DNA, as described previously (68). The following plasmids were used for this study, expressing host and viral genes under the control of a CMV promoter: pcDNA3/-E1B-55K/-E4orf6, pcDNA3/-6×His-Ubiquitin (27, 69), pLenti6.3-V5/-Apobec3A-V5, and pEYFP/-Apobec3A (27). The transfection of the HBV genome was performed with bacmid pHBV1.1, leading to the expression of HBV pgRNA from a CMV promoter; transfection results in HBV infection (70). Luciferase reporter constructs used Renilla or corresponding HAdV promoters (E1A, E2E, MLP; major late promoter) to drive the expression of the promoterless firefly luciferase gene in the pGL3-basic backbone (Promega, E1751). The transfection and experimental set-up were performed as described previously (71), with the siRNA against Apobec3A (200315) and Silencer Select Negative Control (4390843) having been purchased from Invitrogen. Using the RNAiMAX transfection reagent (Thermo Fisher), according to the supplier’s protocol, HepaRG were seeded to subconfluency and were transfected for 24 h before infection.

### Viruses.

Cells were infected with wild-type (wt) HAdV-C5 parental virus (H5*pg*4100), E1B-55K-virus (H5*pm*4149) and E4orf6 HAdV (H5*pm*4154) with a MOI (multiplicity of infection) of 50 (viruses kindly provided by Thomas Dobner/Heinrich Pette Institute, Leibniz Institute of Experimental Virology, Hamburg/Germany) (72). The propagation and the titration of the viruses were performed in HEK293 cells. An immunofluorescence analysis was performed to determine the virus yield by staining for HAdV E2A (73).

### Protein analyses and antibodies.

For the protein analysis, cell lysates were prepared using RIPA buffer as previously described (74), incubated for 30 min on ice, sonicated, and centrifuged at 11.000× rpm/4°C to pellet the insoluble fraction. A Bradford assay (75) was performed to equalize the protein amounts. Immunoprecipitation was performed as described previously (76). An Apobec3A immunoprecipitation dimerization assay was done using a GFP-Trap (ChromoTek) analysis, which was performed according to the manufacturer’s description. For the Western blotting and immunofluorescence, proteins were detected using primary antibodies to directly detect E1B-55K (2A6) (77), E4orf6 (RSA3) (78), and Apobec3A HPA043237 (Atlas Antibodies) as well as to indirectly detect Apobec3A via the V5-tag-recognizing antibody ab27671 (Abcam). The primary GFP antibody ab290 (Abcam) was used to detect YFP-tagged Apobec3A. The monoclonal mouse antibody against β-actin AC-15 (A5441, Sigma-Aldrich), E1A M73 (sc-25, Santa Cruz Biotechnology) (79), E2A B6-8 (80), His (631213, Clontech), p53 (DO-1 sc-126, Santa Cruz Biotechnology), Mre11 (NB100-142, Novus Biologicals), PML (NB100-59787; Novus Biologicals), HBV core (Santa Cruz), rat monoclonal HA (F310), and polyclonal rabbit serum against HAdV capsid L133 (73) were included in this study. The anti-mouse IgG, anti-rat IgG, and anti-rabbit IgG (Jackson/Dianova) were used as secondary antibodies, and these are conjugated to horseradish peroxidase to detect the proteins via chemiluminescence.

### Viral RNA and DNA synthesis.

Viral RNA was isolated from cells and reverse transcribed as described previously (81). Quantitative RT-PCR was performed in a LightCycler 480 (Roche), using 4 μL of 1/10 diluted cDNA, 10 pmol/μL of the corresponding oligonucleotide primers, and 5 μL of SYBR Green Master Mix (Roche) per sample. The following PCR conditions were used: 10 min at 95°C and 40 cycles of 30 s at 95°C, 30 s at 62°C, and 30 s at 72°C. The Apobec and viral mRNA levels that were obtained from triplicate reactions were calculated in relation to levels of the cellular TBP, 18S, or RNA-Polymerase-II mRNA. The following primers were used (fwd, rev): E1A (GTGCCCCATTAACCAGTTG, GGCGTTTACAGCTCAAGTCC), E4orf6 (CCCTCATAAACACGCTGGAC, GCTGGTTTAGGATGGTGGTG), Hexon (CGCTGGACATGACTTTTGAG, GAACGGTGTGCGCAGGTA), 18S (CGGCTACCACATCCAAGGAA, GCTGGAATTACCGCGGCT), RNA-Polymerase-II (GCACCACGTCCAATGACAT, GTCGGCTGCTTCCATAA), TBP (TATAATCCCAAGCGGTTTGC, CTGTTCTTCACTCTTGGCTCCT) Apobec3A (CTACAGGGTCACTTGGTTCATC, CAGTCTCACGTGTGTGTTCTC), Apobec3B (CGCCAGACCTACTTGTGCTA, GCCACAGAGAAGATTCTTAGCC), Apobec3C (TCAACTGCAAGGACGCTGT, ATTGCCTTCATCGGGTTTCT) Apobec3DE (ACCCAAACGTCAGTCGAATC, CACATTTCTGCGTGGTTCTC), Apobec3F (GCCTATGGTCGGAACGAAA, TGGGTCTCAGGATCCACCT), Apobec3G (CCGAGGACCCGAAGGTTAC, TCCAACAGTGCTGAAATTCG), Apobec3H (AGCTGTGGCCAGAAGCAC, CGGAATGTTTCGGCTGTT), Apobec1 (AGGGACCTTGTTAACAGTGGAG, CCAGGTGGGTAGTTGACAAAA) Apobec2 (GGAGAAGTTGGCAGACATCC, TGGCTGTACATGTCATTGCTG), Apobec4 (TTCTAACACCTGGAATGTGATCC, TTTACTGT CTTCTAGCTGCAAACC), and AICDA (GACTTTGG TTATCTTCGCAATAAGA, AGGTCCCAGTCCGAGATGTA).

To analyze the viral DNA, protein lysates were digested with proteinase K for 1 h at 55°C and were then boiled for 10 min at 95°C to inactivate the proteinase K. The PCRs were carried out as follows: 2 min at 95°C, followed by 25 cycles of 1 min at 95°C, 30 s at 57°C, and 15 s at 72°C, followed by 10 min at 72°C and cooling down to 4°C in a final step. The following primers were used (fwd, rev): E1B-55K (ATGAGCGACGAAGAAACCCATCTGAGC, CGGTGTCTGGTCATTAAGCT), E4orf6 (GGAGGATCATCCGCTGCTG, GCACAACACAGGCACACG), and 18S (CGGCTACCACATCCAAGGAA, GCTGGAA TTACCGCGGCT).

### Indirect immunofluorescence.

Immunofluorescence studies were carried out as described previously (46). The experiments were performed in triplicate, and within one experiment, *n* ≥ 3 images were obtained. A Nikon TiE microscope, including an UltraView Vox System (Perkin Elmer), was used to obtain digital images, which were analyzed with the software Volocity 6.2.1 (Perkin Elmer) and were further edited using Adobe Photoshop CS5 and MS Powerpoint.

### SUMO analysis with Ni-NTA assays.

Nickel-NTA (Ni-NTA) assays were performed as described previously (81). For the SUMOylation studies, HepaRG-His-SUMO2 and HepaRG-His/HA cells were transfected and/or infected. For the ubiquitinylation assays, pcDNA3-6×His-ubiquitin was transfected together with the vector of interest and/or was subsequently infected. Briefly, following the harvesting of the cells, the lysates were incubated overnight with HisPur Ni-NTA Resin (Thermo Scientific, 88221) to affinity purify polyhistidine (6×His)-tagged fusion proteins. The His-conjugated resin was stepwise washed under pH-reducing conditions, and the His-conjugates were eluted from the beads via boiling in elution buffer. The samples were then analyzed via SDS-PAGE and Western blotting.

### Interferon assays.

Subconfluent A549 cells were treated with 1,000 U/mL human IFN-α2b (SRP4595, Sigma-Aldrich) 24 h prior to infection with HAdV wt. The cells were harvested at 48 hpi for mRNA isolation, cDNA transcription, and a qPCR analysis for Apobec3A transcription, while a control setup was treated and infected equally to harvest the interferon secreted supernatant at 72 h post treatment. This time point was chosen to exclude potentially remaining interferon from the initial treatment. The dilution rows of supernatant were added for 6 h to the reporter cell line HL116, which expressed a stably integrated luciferase gene under the control of the interferon-responsive promoter region 6 to 16 (36). Dual luciferase reporter assays were performed to measure the interferon secretion, and the median values were calculated.

### Differential DNA denaturing PCR (3D-PCR) analyses for deamination assays.

DNA deaminated by C-to-U(T) conversions contains more AT bases, which reduces the number of hydrogen bonds in the sequence. Thus, deaminated DNA can be selectively detected at lower denaturing temperatures (43).

HepaRG-TR-UGI were induced by 5 μg/mL tetracycline for UGI expression. After the transfection and infection of the cells, DNA was harvested at 72 h postinfection (hpi) using a NucleoSpin Tissue Kit (Machery Nagel) for DNA extraction. 3D-PCR was performed using denaturing temperatures that ranged from 79.5 to 89°C and the primers (fwd, rev) for the HAdV Hexon gene (GCTTCATCCCATTCGCAAGG, CGCGCCACCGAGACGTAC) or HBV cccDNA (ATGGCTGCTARGCTGTGCTGCCAA, AAGTGCACACGGTYYGGCAGAT).

### Cell viability assays.

MTT assays were carried out as described previously (82). Cell viability was further investigated via living cell counting using Trypan blue.

### Dinucleotide analyses.

To investigate the occurrence of dinucleotides, the online tool compseq of Emboss was used, which calculates the observed frequency versus the expected frequency of dinucleotides in a sequence.

### Statistical analysis.

Statistical significance was analyzed by performing unpaired *t* tests or Wilcoxon rank tests if the data were not distributed normally.
